# Identification of Citri Reticulatae Pericarpium (Chenpi) From Different Cultivars via LC–MS/MS and UPLC Coupled With Multivariate Chemometrics Analysis

**DOI:** 10.1002/fsn3.71591

**Published:** 2026-03-06

**Authors:** Danlin Lin, Jinju Zhang, Mingqi Chen, Chuchu Zhong, Hui Cao, Zhiguo Ma, Menghua Wu, Ying Zhang

**Affiliations:** ^1^ Research Center for Traditional Chinese Medicine of Lingnan (Southern China) Jinan University Guangzhou China; ^2^ College of Pharmacy Jinan University Guangzhou China; ^3^ Guangdong Key Lab of Traditional Chinese Medicine Information Technology Guangzhou China; ^4^ State Key Laboratory of Bioactive Molecules and Druggability Assessment Jinan University Guangzhou China

**Keywords:** chemometrics, *Citrus reticulata* Blanco, discrimination, LC–MS/MS, UPLC

## Abstract

Citri Reticulatae Pericarpium (CP), derived from the peel of *Citrus reticulata* Blanco and its cultivated varieties, is widely used and has substantial commercial value. However, notable differences in origin, quality, and price are observed among various CP cultivars. To ensure effective quality control and safe clinical use, a reliable and efficient method for distinguishing these cultivars is urgently needed. This study aimed to investigate the chemical profile differences among seven CP cultivars and to establish a rapid and accurate multi‐component quantitative method using ultra‐high‐performance liquid chromatography (UPLC) for their differentiation. Multivariate statistical analyses, including PCA, OPLS‐DA, and HCA, were employed to distinguish the seven cultivars. LC–MS/MS analysis identified 53 compounds, including 48 flavonoids, and nine representative flavonoids were selected for quantitative determination. The developed UPLC‐based method exhibited high specificity, linearity, precision, repeatability, stability, and accuracy. PCA revealed that *C. reticulata* “Chachi” (GCP), *C. reticulata* “Succosa” (BDZ), *C. reticulata* “Tankan” (JG), and *C. reticulata* “Unshiu” (WZMG) formed distinct clusters, indicating substantial differences in flavonoid composition. In contrast, *C. reticulata* “Kinokuni” (NFMJ), *C. reticulata* “Ponkan” (PG), and *C. reticulata* “Dahongpao” (DHP) grouped together but were further differentiated by PCA and OPLS‐DA, consistent with the HCA results. Hesperetin was quantified exclusively in GCP, suggesting its potential as a cultivar‐specific marker. Although the overall chemical profiles of the seven CP cultivars were similar, their flavonoid contents varied significantly. The combined UPLC quantification and multivariate analyses provide an effective approach for distinguishing major CP cultivars and offer new insights for quality control and future research.

## Graphical abstract

A UPLC‐based quantitative analysis combined with multivariate chemometric methods differentiated seven Citri Reticulatae Pericarpium cultivars based on significant variations in nine flavonoids. Chemometric analysis effectively separated the seven cultivars, and hesperetin was identified as a unique marker for *C. reticulata* “Chachi”. This integrated analytical approach provides a rapid and reliable strategy for cultivar identification and quality control.

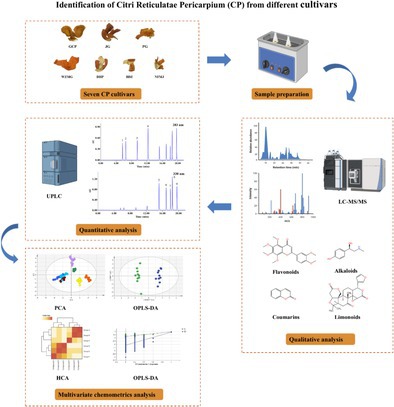

## 1 Introduction

Citri Reticulatae Pericarpium (referred to as “Chenpi” in Chinese, CP) is the dried peel of *Citrus reticulata* Blanco or its cultivars (National Pharmacopeia Committee Commission 2025). It has been reported to possess diverse therapeutic effects in treating respiratory and digestive disorders (Fang et al. 2023; Feng et al. 2020). Modern studies have shown that CP exhibits significant antioxidant (Shi et al. 2024), anticancer (Ahn et al. 2017), anti‐obesity (Zhang, Zhang, et al. 2021), and antibacterial properties (Lin et al. 2021). Volatile oils and flavonoids, including flavonoid glycosides and polymethoxy flavonoids (PMFs), are the primary active components in CP. These components not only contribute to its distinctive aroma but also enhance its overall pharmacological efficacy (Liu et al. 2021).

A wide variety of *Citrus reticulata* cultivars serve as sources of CP. The 2025 edition of the Chinese Pharmacopeia records four major cultivars, including *C. reticulata* “Chachi” (GCP), *C. reticulata* “Dahongpao” (DHP), *C. reticulata* “Unshiu” (WZMG), and *C. reticulata* “Tangerina” (FJ). Among these, GCP specifically refers to *C. reticulata* “Chachi” from Guangdong Province, mainly produced in the Xinhui region (Peng et al. 2024). Herbological studies indicate that GCP has been used since the Yuan Dynasty, nearly one thousand years ago (Yu et al. 2018). Traditionally, GCP is aged for more than 3 years before use, a process believed in Chinese medicine to enhance efficacy and reduce side effects (Zhou et al. 2021). Owing to its superior quality, GCP commands a substantially higher market price (Li et al. 2025). However, its geographically restricted origin limits production, which cannot meet the growing market demand. DHP and WZMG, mainly produced in Sichuan and Zhejiang Provinces, respectively, are more commonly available on the market. According to our previous market provenance survey, FJ has become increasingly scarce due to declining cultivation. The survey also revealed that several other citrus cultivars are used in CP production, including *C. reticulata* “Succosa” (BDZ), *C. reticulata* “Kinokuni” (NFMJ), *C. reticulata* “Tankan” (JG), and *C. reticulata* “Ponkan” (PG). These cultivars may differ significantly in chemical composition, which directly impacts their medicinal properties and nutritional value (Batista et al. 2023; Li et al. 2019). Whether all CP types are suitable for medicinal use remains unclear. Therefore, establishing a reliable method to distinguish major CP cultivars on the market is essential for their quality control and safe clinical application.

Traditionally, CPs can be differentiated based on morphological characteristics. Fresh fruit peels are relatively easy to identify. For example, DHP has a reddish hue, WZMG appears more yellowish, GCP tends to be thicker, and NFMJ is notably thinner. However, these morphological characteristics often disappear after the sun‐drying process. In addition, the peels are cut into small pieces before clinical use, making visual identification even more difficult. DNA‐based methods have also been explored, but due to the complex hybridization patterns of citrus species, accurate identification remains challenging (Li et al. 2019). Luo et al. (2018) reported significant differences in five flavonoid components between GCP and other CP cultivars based on quantitative and statistical analyses. In addition, several studies have applied non‐targeted metabolomics combined with multivariate statistical analyses for cultivar identification (Wang, Wang, et al. 2024; Wang, Lin, et al. 2024; Wang et al. 2025; Yang et al. 2025). However, this approach is limited by high experimental costs, lengthy sample preparation, and complex data processing. Moreover, most existing studies focus on differentiating GCP from other CP cultivars, while methods for distinguishing among non‐GCP cultivars remain insufficiently developed. Thus, a simple and effective classification method is urgently needed to ensure CP quality. In this study, we analyzed the chemical profiles of CP from seven cultivars using LC–MS/MS. Based on the results, nine compounds were selected to develop a simplified UPLC‐based quantitative method. Combined with PCA, OPLS‐DA, and HCA, this approach enables accurate differentiation among the seven CP cultivars commonly found on the market. The results may offer a valuable reference for the quality control and rational clinical use of different CP cultivars.

## 2 Experimental

### 2.1 Materials and Reagents

**FIGURE 1:**
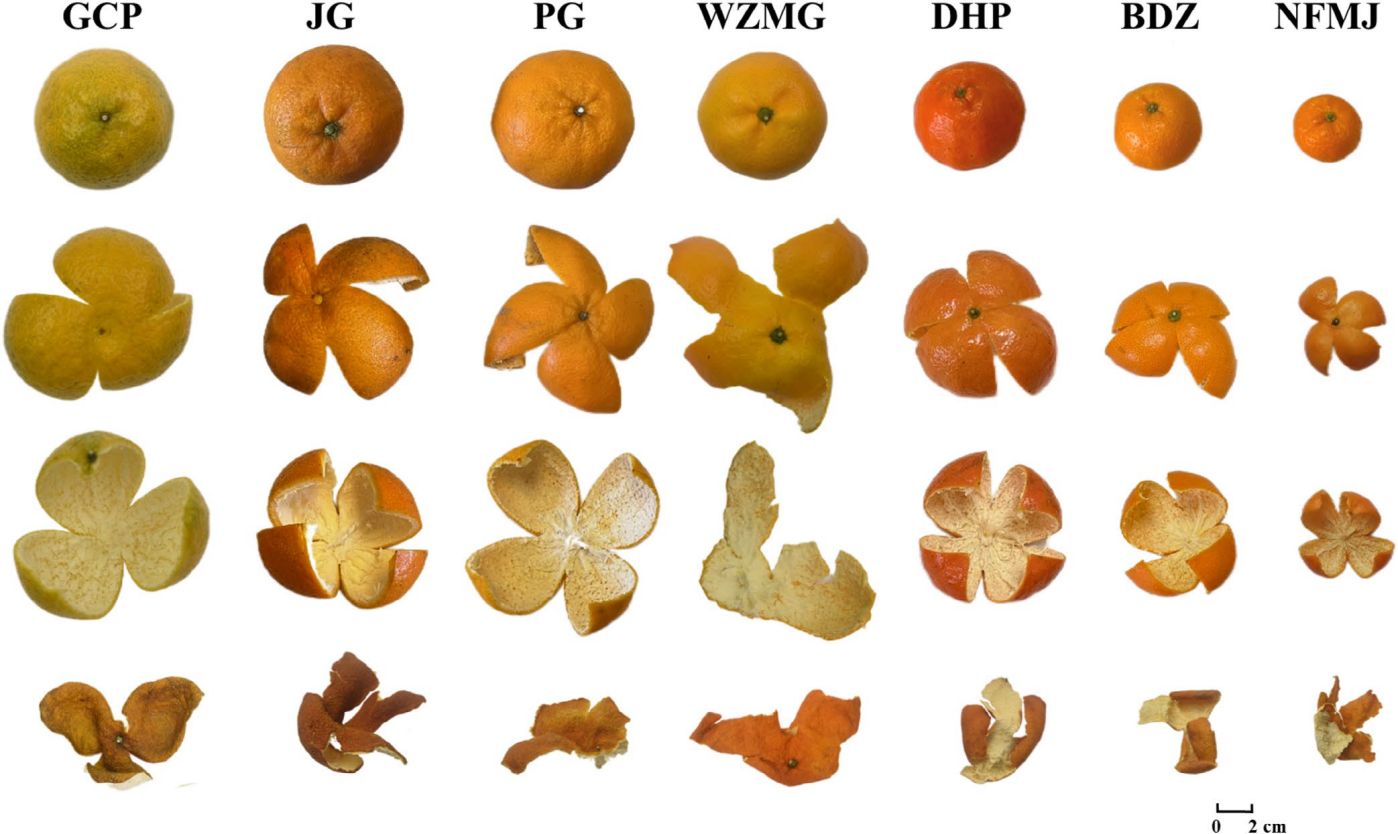
Representative images of the collected samples from the seven CP cultivars.

Seventy batches of CP samples (ten batches each of BDZ, NFMJ, DHP, JG, PG, GCP, and WZMG) were collected. GCP samples were collected from Xinhui, Guangdong Province, China, while the remaining samples were prepared from freshly harvested fruits of the corresponding cultivars. The pericarps were manually peeled and sun‐dried. All samples were authenticated by Prof. Zhiguo Ma of the College of Pharmacy, Jinan University. Voucher specimens were deposited in the Herbarium of Traditional Chinese Medicine at Jinan University. Detailed sample information is presented in Table 1, and representative images of the samples are shown in Figure 1.

**TABLE 1:** Detailed information on the collected samples of the seven CP cultivars.

Sample code	Source	Common name	Scientific name
BDZ‐01	Huangyan District, Taizhou City, Zhejiang Province	Bendizao	*C. reticulata* “Succosa”
BDZ‐02	Yichang City, Hubei Proxince		
BDZ‐03	Huangyan District, Taizhou City, Zhejiang Province		
BDZ‐04	Huangyan District, Taizhou City, Zhejiang Province		
BDZ‐05	Huangyan District, Taizhou City, Zhejiang Province		
BDZ‐06	Huangyan District, Taizhou City, Zhejiang Province		
BDZ‐07	Huangyan District, Taizhou City, Zhejiang Province		
BDZ‐08	Huangyan District, Taizhou City, Zhejiang Province		
BDZ‐09	Huangyan District, Taizhou City, Zhejiang Province		
BDZ‐10	Huangyan District, Taizhou City, Zhejiang Province		
NFMJ‐01	Nanfeng County, Fuzhou City, Jiangxi Province	Nanfengmiju	*C. reticulata* “Kinokuni”
NFMJ‐02	Nanfeng County, Fuzhou City Jiangxi Province		
NFMJ‐03	Fuzhou City, Jiangxi Province		
NFMJ‐04	Nanfeng County, Fuzhou City, Jiangxi Province		
NFMJ‐05	Nanfeng County, Fuzhou City, Jiangxi Province		
NFMJ‐06	Nanfeng County, Fuzhou City, Jiangxi Province		
NFMJ‐07	Nanfeng County, Fuzhou City, Jiangxi Province		
NFMJ‐08	Nanfeng County, Fuzhou City, Jiangxi Province		
NFMJ‐09	Nanfeng County, Fuzhou City, Jiangxi Province		
NFMJ‐10	Nanfeng County, Fuzhou City, Jiangxi Province		
DHP‐01	Anyue County, Ziyang City, Sichuan Province	Dahongpao	*C. reticulata* “Dahongpao”
DHP‐02	Anyue County, Ziyang City, Sichuan Province		
DHP‐03	Zigong City, Sichuan Province		
DHP‐04	Wanzhou District, Chongqing City		
DHP‐05	Wanzhou District, Chongqing City		
DHP‐06	Ziyang City, Sichuan Province		
DHP‐07	Renshou County, Meishan City, Sichuan Province		
DHP‐08	Wanzhou District, Chongqing City		
DHP‐09	Wanzhou District, Chongqing City		
DHP‐10	Wanzhou District, Chongqing City		
JG‐01	Jieyang City, Guangdong Province	Jiaogan	*C. reticulata* “Tankan”
JG‐02	Chaozhou City, Guangdong Province		
JG‐03	Jieyang City, Guangdong Province		
JG‐04	Chaozhou City, Guangdong Province		
JG‐05	Chaozhou City, Guangdong Province		
JG‐06	Chaozhou City, Guangdong Province		
JG‐07	Zhangzhou City, Fujian Province		
JG‐08	Shantou City, Guangdong Province		
JG‐09	Zhangzhou City, Fujian Province		
JG‐10	Jieyang City, Guangdong Province		
PG‐01	Qianwei County, Leshan City, Sichuan Province	Penggan	*C. reticulata* “Ponkan”
PG‐02	Zizhong County, Neijiang City, Sichuan Province		
PG‐03	Longquanyi District, Chengdu City, Sichuan Province		
PG‐04	Mianyang City, Sichuan Province		
PG‐05	Leshan City, Sichuan Province		
PG‐06	Yongchun County, Quanzhou City, Fujian Province		
PG‐07	Yongchun County, Quanzhou City, Fujian Province		
PG‐08	Yongchun County, Quanzhou City, Fujian Province		
PG‐09	Yongchun County, Quanzhou City, Fujian Province		
PG‐10	Yong'an City, Sanming City, Fujian Province		
GCP‐01 (aging 1 year)	Xinhui City, Guangdong Province	Chazhigan	*C. reticulata* “Chachi”
GCP‐02 (aging 2 year)	Xinhui City, Guangdong Province		
GCP‐03 (aging 3 year)	Xinhui City, Guangdong Province		
GCP‐04 (aging 3 year)	Xinhui City, Guangdong Province		
GCP‐05 (aging 3 year)	Xinhui City, Guangdong Province		
GCP‐06 (aging 4 year)	Xinhui City, Guangdong Province		
GCP‐07 (aging 4 year)	Xinhui City, Guangdong Province		
GCP‐08 (aging 5 year)	Xinhui City, Guangdong Province		
GCP‐09 (aging 5 year)	Xinhui City, Guangdong Province		
GCP‐10 (aging 5 year)	Xinhui City, Guangdong Province		
WZMG‐01	Linhai City, Taizhou City, Zhejiang Province	Wenzhoumigan	*C. reticulata* “Unshiu”
WZMG‐02	Huangyan District, Taizhou City, Zhejiang Province		
WZMG‐03	Huangyan District, Taizhou City, Zhejiang Province		
WZMG‐04	Linhai City, Taizhou City, Zhejiang Province		
WZMG‐05	Huangyan District, Taizhou City, Zhejiang Province		
WZMG‐06	Huangyan District, Taizhou City, Zhejiang Province		
WZMG‐07	Shimen County, Changde City, Hunan Province		
WZMG‐08	Shimen County, Changde City, Hunan Province		
WZMG‐09	Shimen County, Changde City, Hunan Province		
WZMG‐10	Shimen County, Changde City, Hunan Province		

Chemical standards (purity > 97%) of narirutin, hesperidin, didymin, hesperetin, sinensetin, nobiletin, 3′,4′,3,5,6,7,8‐heptamethoxyflavone, tangeretin, and 5‐demethylnobiletin were obtained from Ruifensi Biotechnology Co. Ltd. (Chengdu, China). Acetonitrile (ACN) and methanol were purchased from Thermo Fisher Scientific (Waltham, MA, USA). Ultrapure water was produced using a Milli‐Q water purification system (Bedford, MA, USA), and HPLC‐grade acetic acid was purchased from Macklin Biochemical Co. Ltd. (Shanghai, China).

### 2.2 LC–MS/MS Analysis

#### 2.2.1 Sample Preparation

Sample preparation methods were optimized by evaluating the extraction methods, extraction time, and solvent type. All samples were ultimately processed using the same protocol for both LC–MS/MS and UPLC analyses. Briefly, the samples were crushed and passed through a 24‐mesh sieve. Approximately 0.4 g of powdered material was accurately weighed and extracted with 50 mL of methanol using heated reflux for 1 h. The resulting extract was then filtered through a 0.22 μm membrane filter. Two independent replicates were prepared for each sample. Blank control samples were prepared following the same procedure but without adding any sample material.

#### 2.2.2 UPLC‐Q‐Exactive Orbitrap MS Analysis

LC–MS/MS analysis was performed using a Q‐Exactive Orbitrap mass spectrometer (Thermo Fisher Scientific, Waltham, MA, USA) coupled with a Thermo Vanquish UPLC (Thermo Fisher Scientific, Waltham, MA, USA). Chromatographic separation was carried out on a Waters Acquity UPLC BEH C18 column (2.1 × 100 mm, 1.7 μm; Waters Corp., Milford, MA, USA). The mobile phase consisted of solvent A (0.1% formic acid in water) and solvent B (ACN). The gradient elution program was as follows: 0–2 min, 95% B; 2–42 min, 95%–5% B; 42–47 min, 5% B; 47–47.1 min, 5%–95% B; 47.1–50 min, 95% B. The flow rate was 0.3 mL/min, the injection volume was 3 μL, and the column temperature was maintained at 40°C.

Mass spectrometry parameters were set as follows: positive ion mode, spray voltage of 3.5 kV, sheath gas flow rate of 35 arb, auxiliary gas flow rate of 10 arb, and probe heater temperature of 350°C. Full MS scans were acquired at a resolution of 70,000 with an AGC target of 1e^6^ and a scan range of 100–1500 m/z. For dd‐MS^2^ acquisition, the resolution was set to 17,500 with an AGC target of 2e^5^ and an isolation width of 1.5 m/z.

#### 2.2.3 Data Processing

Raw LC–MS/MS data files were imported into the Compound Discoverer software (version 3.0; Thermo Fisher Scientific, Waltham, MA, USA) for peak detection, alignment, and normalization. Compound identification was performed using the integrated mzCloud and mzVault databases, and further confirmed by comparison with compounds previously reported in citrus species. Compounds showing substantial differences in peak areas among different cultivars and for which reference standards were available were selected to establish a multi‐component UPLC‐based quantitative method for differentiating CP cultivars.

### 2.3 UPLC Analysis

A Waters ACQUITY UPLC system (Waters Corp., Milford, MA, USA) was employed to quantify nine flavonoids. Chromatographic separation was achieved using a Waters Acquity UPLC HSS T3 column (2.1 × 100 mm, 1.8 μm) with detection by a photodiode array detector. The detection wavelengths were set at 283 nm for narirutin, hesperidin, didymin, and hesperetin, and 330 nm for sinensetin, nobiletin, 3′,4′,3,5,6,7,8‐heptamethoxyflavone, tangeretin, and 5‐demethylnobiletin. The mobile phase consisted of solvent A (0.1% formic acid in water) and solvent B (ACN). The gradient program was: 0–12 min, 15%–45% B; 12–16 min, 45%–65% B; 16–20 min, 65%–15% B; and 20–22 min, 15% B. The flow rate was 0.3 mL/min, the injection volume was 2.0 μL, and the column temperature was maintained at 30°C.

### 2.4 Standard Solution Preparation

Nine reference standards were accurately weighed, dissolved in methanol, and serially diluted to prepare working solutions. The concentration ranges were as follows: 0.9950–59.70 μg/mL for narirutin, 20.05–1203.00 μg/mL for hesperidin, 0.5325–31.95 μg/mL for didymin, 0.07575–4.5450 μg/mL for hesperetin, 0.5025–30.15 μg/mL for sinensetin, 1.52–91.20 μg/mL for nobiletin, 0.2675–16.05 μg/mL for 3′,4′,3,5,6,7,8‐heptamethoxyflavone, 1.07–64.20 μg/mL for tangeretin, and 0.2625–15.75 μg/mL for 5‐demethylnobiletin.

### 2.5 UPLC Method Validation

#### 2.5.1 Calibration Curves, Limits of Detection (LOD), and Limits of Quantification (LOQ)

Calibration curves for each compound were constructed by plotting peak area (*y*) against concentration (*x*). LOD and LOQ were determined based on signal‐to‐noise ratios (S/N) of 3 and 10, respectively.

#### 2.5.2 Precision, Stability and Repeatability

Intraday precision was evaluated using six replicate injections within a single day, while interday precision was assessed over three consecutive days. Sample stability was examined by analyzing DHP‐01 at 0, 1, 2, 4, 8, 12, and 24 h. Repeatability was assessed using six independently prepared replicates of DHP‐01 and GCP‐07. Relative standard deviations (RSDs) of peak areas or compound concentrations for the nine flavonoids were calculated to evaluate precision, stability, and repeatability.

#### 2.5.3 Recovery

Recovery was evaluated using the standard addition method. Known amounts of standards were added to DHP‐01 and GCP‐07 at 80%, 100%, and 120% of their original concentrations. Recovery rates were calculated based on differences in compound concentrations before and after spiking.

### 2.6 Data Analysis

PCA and OPLS‐DA were performed using SIMCA 14.1 software (Umetrics AB, Umeå, Sweden) based on the quantified contents of nine flavonoids to differentiate the seven CP cultivars. HCA was conducted using Ward's method and squared Euclidean distance on an online platform (https://www.bioinformatics.com.cn) to classify the cultivars according to their flavonoid content.

## 3 Results and Discussion

### 3.1 Identification of Chemical Components

As shown in Appendix S1, the representative total ion chromatograms of the seven CP cultivars exhibited high overall similarity, while subtle differences reflected variations in their chemical profiles. As summarized in Table 2, a total of 53 compounds were identified across the seven cultivars, including 48 flavonoids, 2 alkaloids, 2 limonoids, and 1 coumarin. These results indicate that flavonoids are the predominant constituents in CP. Previous studies have demonstrated that glycosylated flavanones and polymethoxylated flavones represent the major bioactive forms among these flavonoids. In this study, several representative compounds were identified, including hesperidin, naringin, sinensetin, nobiletin, and tangeretin. This result is consistent with previous reports on CP metabolites (Munir et al. 2024; Wang, Wang, et al. 2024; Wang, Lin, et al. 2024). Nine compounds that exhibited the greatest variation among the seven CP cultivars were selected for subsequent UPLC‐based quantitative analysis. These compounds included narirutin, hesperidin, didymin, hesperetin, sinensetin, nobiletin, 3′,4′,3,5,6,7,8‐heptamethoxyflavone, tangeretin, and 5‐demethylnobiletin.

**TABLE 2:** Compounds identified in the seven CP cultivars by LC–MS/MS.

No.	Name	Formula	[M + H]^+^ (m/z)	ppm	Fragment ions (m/z)
**Flavonoids**					
1	5,7‐Dihydroxy‐3‐(4‐hydroxyphenyl)‐4H‐chromen‐4‐one	C_15_H_10_O_5_	271.05955	−2.02	271.05939, 153.01801, 119.04911, 272.06274, 171.02814
2	Butein	C_15_H_12_O_5_	273.07533	−1.56	273.07480, 255.06459, 147.04367, 119.04907, 91.05455
3	(2E)‐3‐(3,4‐Dihydroxy‐2‐methoxyphenyl)‐1‐(4‐hydroxyphenyl)prop‐2‐en‐1‐one	C_16_H_14_O_5_	287.09096	−1.55	269.07999, 245.07996, 227.06981, 199.07523, 181.06438
4	Eriodictyol	C_15_H_12_O_6_	289.07022	−1.55	153.01793, 289.07004, 163.03867, 171.02844, 135.04376
5	5‐Hydroxy‐6,7‐dimethoxyflavone	C_17_H_14_O_5_	299.0909	−1.68	284.06717, 256.07236, 241.04913, 170.02104, 124.01573
6	Tectorigenin	C_16_H_12_O_6_	301.07011	−1.38	286.04620, 258.05258, 245.07887, 210.33113, 139.01897
7	Morin	C_15_H_10_O_7_	303.04944	−1.61	285.03891, 229.04909, 137.02315, 111.00779, 95.04935
8	5,7‐Dihydroxy‐2‐(2,3,4‐trihydroxyphenyl)‐4H‐chromen‐4‐one	C_15_H_10_O_7_	303.04943	−1.66	285.03827, 257.04379, 229.04901, 177.05415, 153.01784
9	5,7‐Dihydroxy‐2‐(4‐hydroxy‐3‐methoxyphenyl)‐3,4‐dihydro‐2H‐1‐Benzopyran‐4‐one	C_16_H_14_O_6_	303.08566	−2.18	270.05170, 25,304,904, 177.05415, 171.02831, 153.01782
10	Hesperetin^a^	C_16_H_14_O_6_	303.08585	−1.53	303.08536, 179.03343, 177.05420, 149.05951, 117.03361
11	Pectolinarigenin	C_17_H_14_O_6_	315.08568	−2.02	300.06198, 285.03790, 257.04337, 254.05623, 121.02818
12	5,7‐Dihydroxy‐3,8‐dimethoxy‐2‐phenyl‐4H‐chromen‐4‐one	C_17_H_14_O_6_	315.08569	−1.97	285.03851, 257.04382, 254.05666, 153.01768, 121.02834
13	Rhamnetin	C_16_H_12_O_7_	317.0651	−1.52	274.04700, 228.04054,163.03835, 121.02853, 93.03359
14	5‐Hydroxy‐7,3′,4′‐trimethoxyflavone	C_18_H_16_O_6_	329.10135	−1.88	213.03885, 195.02832, 85.02876, 57.03413, 12,502,324
15	Aurantio‐obtusin	C_17_H_14_O_7_	331.08073	−1.52	316.05664, 301.03323, 273.03876, 245.04387, 149.05943
16	Arthone C	C_16_H_12_O_8_	333.05997	−1.58	318.03619, 301.03342, 244.03575, 169.01279, 137.02307
17	6‐Demethoxytangeretin	C_19_H_18_O_6_	343.11678	−2.55	313.06961, 285.07513, 181.01263, 153.01793, 133.06465
18	Eupatilin	C_18_H_16_O_7_	345.0896	−1.35	330.07260, 314.04251, 287.05478, 191.06993, 148.05150
19	Gardenin B	C_19_H_18_O_7_	359.11194	−1.70	359.1116, 326.07764, 298.08270, 227.07010, 93.12201
20	Tangeretin^a^	C_20_H_20_O_7_	373.12714	−2.76	343.08054, 325.02992, 297.07495,271.05945, 183.02855
21	Sinensetin^a^	C_20_H_20_O_7_	373.12716	−2.68	343.08041, 329.10080, 312.09802, 163.07484, 153.01794
22	Casticin	C_19_H_18_O_8_	375.10682	−1.67	345.05991, 327.04913, 302.04105, 230.05812, 169.01297
23	5‐Demethylnobietin^a^	C_20_H_20_O_8_	389.1212	−2.83	359.07458, 341.06396, 215.01770, 197.00722, 169.01242
24	Polydatin	C_20_H_22_O_8_	391.13772	−2.56	241.06987, 211.02307, 183.02831, 165.01793, 155.03354
25	Nobiletin^a^	C_21_H_22_O_8_	403.1369	−4.57	373.08994, 327.08441, 211.02272, 183.02797, 165.05397
26	Puerarin	C_21_H_20_O_9_	417.11712	−2.13	399.10638, 363.0863, 351.08578, 339.08560, 297.07697
27	Neoisoliquiritin	C_21_H_22_O_9_	419.1329	−1.81	389.08563, 361.09088, 328.05682, 303.04904, 165.05423
28	Vitexin	C_21_H_20_O_10_	433.11226	−1.53	415.10114, 397.09106, 313.06973, 283.05923, 121.02833
29	3,3′,4′,5,6,7,8‐Heptamethoxyflavone^a^	C_22_H_24_O_9_	433.14848	−1.92	418.12457, 403.10150, 345.06064, 317.06366, 165.05409
30	Prunin	C_22_H_22_O_10_	435.12772	−1.96	273.07504, 195.02785, 153.01793, 147.04376, 119.04915
31	Homoorientin	C_21_H_20_O_11_	449.10715	−1.53	383.07507, 353.06479, 329.06476, 299.05417, 283.05908
32	Isosakuranin	C_22_H_24_O_10_	449.14331	−2.05	287.09052, 171.02847, 161.05934, 153.01790, 133.06458
33	Obacunone	C_26_H_30_O_7_	455.20565	−1.72	409.19922, 315.13516, 161.05940, 105.07005, 95.04932
34	Homoplantaginin	C_22_H_22_O_11_	463.12257	−1.98	301.06961, 286.04617, 258.05157, 229.04839, 153.01836
35	Isoscoparin	C_22_H_22_O_11_	463.12273	−1.63	427.10123, 367.08026, 343.08032, 313.06967, 183.02798
36	Iridin	C_24_H_26_O_13_	523.14387	−1.43	361.0907, 346.0675, 331.04385, 328.05649, 257.0436
37	Narirutin^a^	C_27_H_32_O_14_	581.18533	−2.00	273.0751, 195.02849, 153.01801, 85.02882, 71.04974
38	Naringin	C_27_H_32_O_14_	581.18549	−1.72	383.11234, 32,906,491, 273.07507, 195.02849, 85.02879
39	Linarin	C_28_H_32_O_14_	593.18542	−1.79	447.12711, 285.07510, 242.05614, 129.05457, 85.02870
40	Vicenin II	C_27_H_30_O_15_	595.16458	−1.96	457.11148, 337.06982, 325.06952, 307.05896, 295.05923
41	4′‐O‐Glucosylvitexin	C_27_H_30_O_15_	595.16469	−1.96	397.09085, 367.08032, 313.06976, 195.02856, 57.03415
42	Didymin^a^	C_28_H_34_O_14_	595.2009	−2.09	287.09030, 195.02859, 153.01779, 85.02872, 71.04970
43	Eriocitrin	C_27_H_32_O_15_	597.18046	−1.57	289.07013, 195.02849, 163.03864, 85.02873, 71.04968
44	2″‐O‐β‐L‐Galactopyranosylorientin	C_27_H_30_O_16_	611.15959	−1.75	395.07321, 353.06458, 329.06543, 311.05420, 299.05460
45	Rutin	C_27_H_30_O_16_	611.15964	−1.69	447.12836, 303.04993, 153.01791, 85.02877, 71.04966
46	Hesperidin^a^	C_28_H_34_O_15_	611.196	−1.72	303.08551, 177.05423, 153.0179, 85.02879, 71.04968
47	Isorhamnetin‐3‐O‐nehesperidine	C_28_H_32_O_16_	625.17528	−1.66	317.06467, 302.04120, 275.05057, 245.04330, 99.04383
48	Methyl hesperidin	C_29_H_36_O_15_	625.2115	−1.96	317.10016, 191.06992, 153.01797, 85.02869, 71.04976
**Alkaloids**					
49	Synephrine	C_9_H_13_NO_2_	168.10167	−1.43	150.09105, 91.05453, 107.04941, 135.06769, 119.04909
50	Diosmin	C_28_H_32_O_15_	609.18038	−1.68	463.12186, 301.06976, 286.04626, 258.05164, 85.02876
**Limonoids**					
51	Limonin	C_26_H_30_O_8_	471.20048	−1.84	425.1944, 409.19894, 367.18973,161.05933, 95.01302
52	Deoxylimonin	C_26_H_30_O_7_	455.20578	−1.44	437.19247, 377.17133, 243.10046, 185.09599, 95.04933
**Coumarins**					
53	Coumarin	C_9_H_6_O_2_	147.04387	−1.30	147.08025, 119.04910, 105.06993, 91.05452, 65.03903

^a^ The compound was selected to establish the UPLC quantification method.

### 3.2 Methods Optimization

To optimize extraction efficiency, several parameters were systematically evaluated, including extraction method (ultrasonic vs. reflux), extraction solvent (50%, 70%, and 100% methanol), extraction time (20, 30, 40, 60, and 90 min), and solvent volume (30, 40, and 50 mL). As shown in Appendix S2A, the reflux method yielded significantly higher extraction efficiency than ultrasonic extraction. Among the solvents tested, 100% methanol generated the highest peak areas and was therefore selected as the optimal extraction solvent (Appendix S2B). Extraction efficiency increased with extraction time and reached a plateau at 60 min (Appendix S2C). Similarly, as the volume of 100% methanol increased, extraction efficiency improved and stabilized at 50 mL (Appendix S2D). Accordingly, extraction with 50 mL of 100% methanol for 60 min was selected as the optimal condition, balancing both efficiency and overall practicality.

To optimize chromatographic separation, different columns (Kinetex EVO C18, Waters ACQUITY UPLC BEH C18, and Waters ACQUITY UPLC HSS T3) and mobile phases (0.1% formic acid‐ACN, 0.1% acetic acid‐ACN, and 0.1% formic acid‐methanol) were compared. The optimized method employed a Waters ACQUITY UPLC HSS T3 column with 0.1% formic acid‐ACN as the mobile phase. Under the optimized chromatographic conditions, all nine target compounds achieved baseline resolution (resolution > 1.5), as demonstrated by the chromatogram of the mixed standard solution. By matching the retention times and UV spectra of sample peaks with those of the reference standards, eight compounds were identified in all seven CP cultivars, whereas hesperetin was detected only in GCP.

**FIGURE 2:**
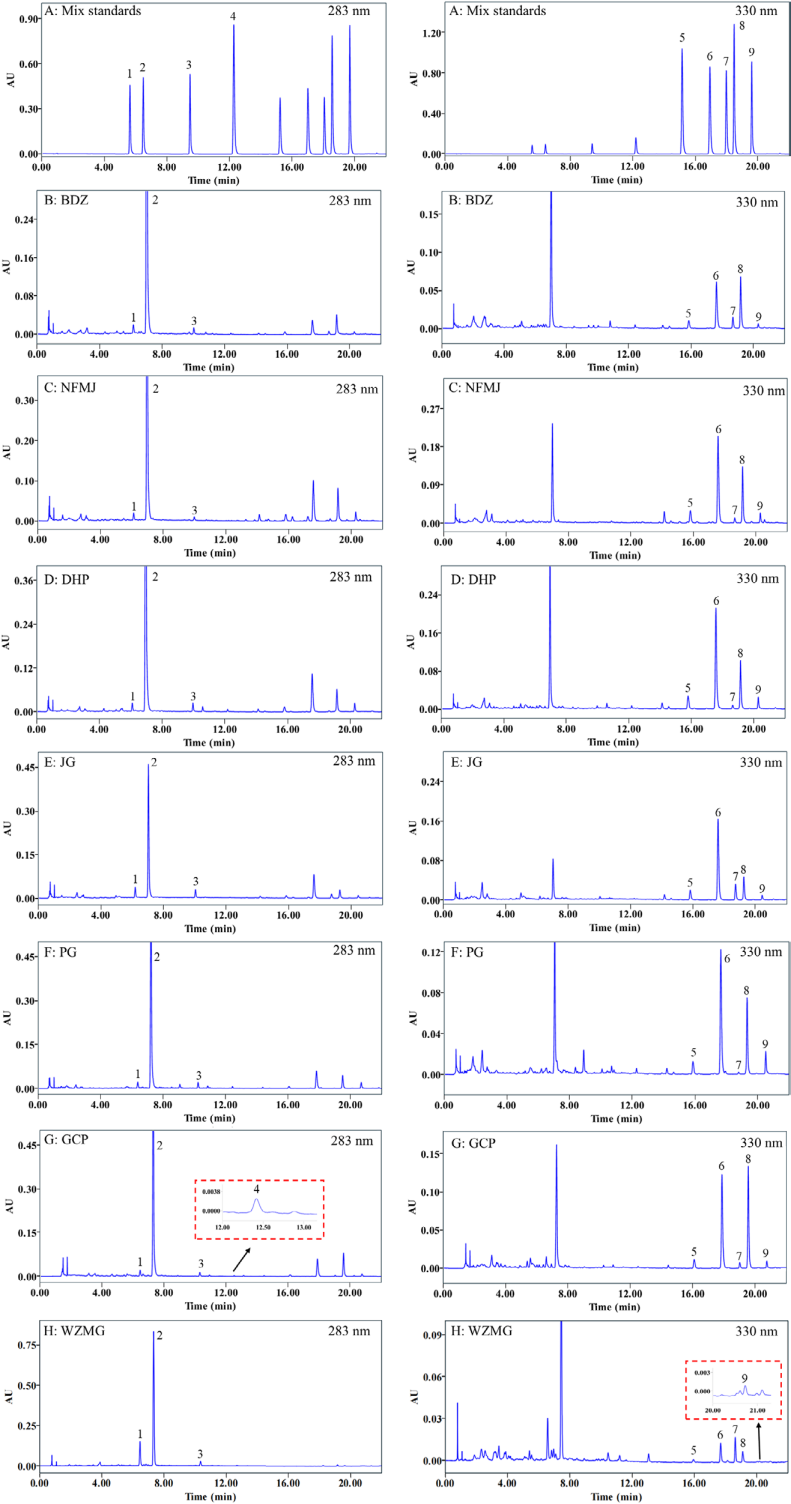
Representative UPLC chromatograms of mixed standards (A) and the seven CP cultivars: BDZ (B), NFMJ (C), DHP (D), JG (E), PG (F), GCP (G), and WZMG (H). Target peaks: (1) Narirutin, (2) Hesperidin, (3) Didymin, (4) Hesperetin, (5) Sinensetin, (6) Nobiletin, (7) 3′,4′,3,5,6,7,8‐Heptamethoxyflavone, (8) Tangeretin, (9) 5‐Demethylnobiletin.

As shown in Figure 2A, all nine standards were well separated and eluted within 22 min. Narirutin, hesperidin, didymin, and hesperetin exhibited strong UV absorbance at 283 nm, whereas sinensetin, nobiletin, 3′,4′,3,5,6,7,8‐heptamethoxyflavone, tangeretin, and 5‐demethylnobiletin showed maximum absorbance at 330 nm. The retention times (Figure 2B–H) and UV spectral patterns (Appendix S3) of the nine target compounds in all samples were consistent with those of the corresponding standards. In addition, all nine target peaks in the sample solutions also achieved baseline resolution (resolution > 1.5). These results demonstrate the good specificity of the established chromatographic method for all nine target compounds.

### 3.3 Methods Validation

As shown in Table 3, the optimized sample preparation and UPLC method exhibited excellent linearity for mixtures of the nine flavonoid standards across the tested concentration ranges (*r* > 0.999), indicating accurate quantification over a broad range. The LODs and LOQs for the nine flavonoids ranged from 0.0379 to 0.152 μg/mL and 0.114 to 0.456 μg/mL, respectively, confirming the high sensitivity of the developed method. Precision assessment showed that the RSDs of peak areas were below 0.88% for intraday and below 0.79% for interday analyses, demonstrating excellent precision. Repeatability, evaluated by analyzing six independently prepared replicates of a single sample, yielded RSD values ranging from 1.06% to 2.05%. Stability testing revealed that the RSDs of peak areas remained below 2.06% over 3 days, indicating good sample stability. Additionally, the method showed high accuracy, with recovery rates ranging from 96.32% to 101.4%.

**TABLE 3:** Validation of the UPLC‐based quantitative method.

Compound	Calibration curve	*r*	Working range (μg/mL)	LOD (μg/mL)	LOQ (μg/mL)	Precision (RSD, %)		Repeatability (RSD, %)	Stability (RSD, %)	Recovery	
						Interday	Intraday			Mean	RSD (%)
Narirutin	*y* = 13720*x* + 2481.9	0.9997	0.995–39.8	0.0995	0.299	0.49	0.88	1.20	0.95	98.26	1.88
Hesperidin	*y* = 10025*x* + 42470	0.9980	20.0–802	0.134	0.401	0.17	0.47	1.12	0.24	97.48	1.99
Didymin	*y* = 18814*x* + 1340	0.9995	0.532–32.0	0.0888	0.266	0.44	0.44	1.08	0.41	98.88	1.91
Hesperetin	*y* = 47448*x* + 459.41	0.9994	0.152–4.54	0.0379	0.114	0.79	0.24	2.05	2.06	99.84	1.26
Sinensetin	*y* = 29525*x* − 3895.5	0.9998	0.502–30.2	0.0838	0.251	0.06	0.73	1.06	0.32	101.4	0.99
Nobiletin	*y* = 24445*x* − 6870.5	0.9998	1.52–91.2	0.152	0.456	0.23	0.38	1.24	0.40	100.1	1.22
3′,4′,3,5,6,7,8‐Heptamethoxyflavone	*y* = 18493*x* − 392.07	0.9994	0.268–16.0	0.0446	0.134	0.03	0.88	1.14	0.31	99.89	2.13
Tangeretin	*y* = 26632*x* − 6131	0.9997	1.07–64.2	0.107	0.321	0.15	0.37	1.32	0.34	96.88	1.14
5‐Demethylnobiletin	*y* = 18796*x* − 1982.6	0.9997	0.262–15.8	0.0438	0.131	0.02	0.82	1.42	0.80	96.32	1.44

Abbreviations: LOD, limit of detection; LOQ, limit of quantification; RSD, relative standard deviation.

### 3.4 Quantification of Nine Flavonoids and Multivariate Analysis

#### 3.4.1 Quantitative Analysis

**FIGURE 3:**
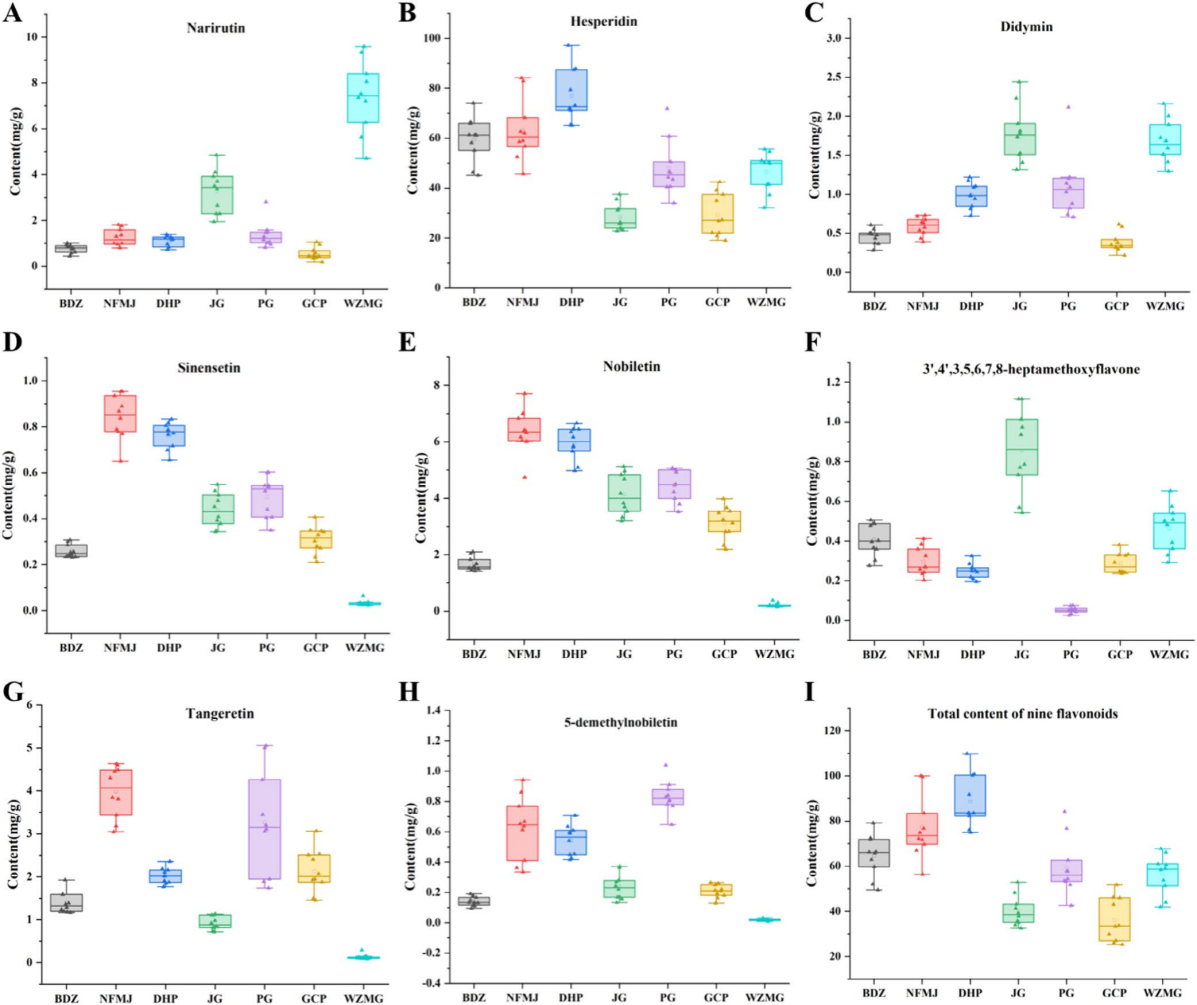
Comparison of the contents of the eight flavonoids (hesperetin was quantified only in GCP) and the total flavonoid content across the seven CP cultivars.

A total of 70 batches of CP samples from seven cultivars were analyzed using the established method. As shown in Table 4 and Figure 3, eight flavonoids, including narirutin, hesperedin, didymin, sinensetin, nobiletin, 3′,4′,3,5,6,7,8‐heptamethoxyflavone, tangeretin, and 5‐demethylnobiletin were detected in all samples, while hesperetin could be quantified only in GCP. The total content of the eight quantified flavonoids (nine in GCP) ranged from 36.24 to 88.64 mg/g across the seven CP cultivars, with DHP showing the highest overall content. Among these compounds, hesperidin was the most abundant, accounting for 71% to 92% of the total flavonoid content of the eight flavonoids. Each cultivar displayed a distinct flavonoid profile. For example, WZMG contained a higher concentration of narirutin but relatively lower levels of polymethoxylated flavones such as sinensetin, nobiletin, tangeretin, and 5‐demethylnobiletin. In contrast, JG had lower hesperidin levels but higher levels of didymin and 3′,4′,3,5,6,7,8‐heptamethoxyflavone. Hesperidin is the predominant flavonoid glycoside in CP, whereas nobiletin and tangeretin are representative polymethoxylated flavones with reported neuroprotection and antitumor activities. In the Chinese Pharmacopeia, hesperidin, nobiletin, and tangeretin are designated as quality markers for CP (National Pharmacopeia Committee Commission 2025). In this study, DHP exhibited the highest hesperidin content, while nobiletin and tangeretin were most abundant in NFMJ. The combined concentration of these three markers was also highest in DHP. Although GCP has traditionally been regarded as the highest‐quality CP, its levels of hesperidin, nobiletin, and tangeretin were significantly lower than those of the other cultivars, and it also exhibited the lowest total content of all nine compounds.

**TABLE 4:** Contents of the nine flavonoids in the seven CP cultivars (*x* ± SD)^a^.

Sample	Content (mg/g)									Total content (mg/g)
	Narirutin	Hesperidin	Didymin	Hesperetin	Sinensetin	Nobiletin	3′,4′,3,5,6,7,8‐Heptamethoxyflavone	Tangeretin	5‐Demethyl nobiletin	
*Citrus reticulata* “Succosa” (BDZ)	0.77 ± 0.17	59.51 ± 8.46	0.46 ± 0.09	—^b^	0.26 ± 0.03	1.67 ± 0.23	0.41 ± 0.08	1.39 ± 0.23	0.14 ± 0.03	64.6
*Citrus reticulata* “Kinokuni” (NFMJ)	1.24 ± 0.36	63.25 ± 11.66	0.58 ± 0.11	—	0.84 ± 0.09	6.36 ± 0.73	0.30 ± 0.07	3.98 ± 0.57	0.62 ± 0.19	77.18
*Citrus reticulata* “Dahongpao” (DHP)	1.08 ± 0.23	77.04 ± 10.09	0.99 ± 0.15	—	0.76 ± 0.05	5.95 ± 0.55	0.25 ± 0.04	2.02 ± 0.17	0.54 ± 0.09	88.64
*Citrus reticulata* “Tankan” (JG)	3.26 ± 0.89	28.39 ± 4.99	1.77 ± 0.34	—	0.44 ± 0.07	4.14 ± 0.68	0.86 ± 0.20	0.92 ± 0.15	0.24 ± 0.08	40.01
*Citrus reticulata* “Ponkan” (PG)	1.37 ± 0.53	47.90 ± 10.44	1.10 ± 0.38	—	0.50 ± 0.08	4.45 ± 0.53	0.052 ± 0.02	3.27 ± 1.15	0.83 ± 0.10	59.46
*Citrus reticulata* “Chachi” (GCP)	0.55 ± 0.26	29.22 ± 8.11	0.38 ± 0.12	0.029 ± 0.02	0.31 ± 0.06	3.12 ± 0.55	0.29 ± 0.05	2.13 ± 0.47	0.21 ± 0.04	36.24
*Citrus reticulata* “Unshiu” (WZMG)	7.41 ± 1.55	46.43 ± 7.84	1.68 ± 0.27	—	0.032 ± 0.01	0.22 ± 0.07	0.46 ± 0.12	0.13 ± 0.059	0.019 ± 0.01	56.38

^a^ Each content is presented as the average value ± standard deviation.

^b^ Not detected or fail quantification.

According to TCM theory, herbs such as CP, Atractylodis Rhizoma, and Aurantii Fructus possess a “dry” nature, which may lead to undesirable effects. Modern research suggests that this “dryness” may be associated with pro‐inflammatory properties (Gao et al. 2021; Shi et al. 2024; Zhang, Zhao, et al. 2021). Therefore, CP is typically aged for at least 3 years before use to mitigate such effects. Notably, GCP is the only cultivar currently subjected to this aging process. Previous studies have demonstrated that chemical composition changes during aging, which may account for the lower flavonoid levels observed in GCP. These results indicate that quality evaluation should not rely solely on the absolute contents of hesperidin, nobiletin, and tangeretin. A more comprehensive quality assessment system may be required.

In addition, hesperetin was quantified only in GCP samples, with an average concentration of 0.029 mg/g, whereas its levels in the other six cultivars were below the LOQ. Peak extraction from LC–MS/MS data confirmed that hesperetin signals were markedly lower in non‐GCP samples, corroborating the quantification results. Given its significantly higher concentration in GCP, hesperetin may serve as a characteristic marker for distinguishing this cultivar. A previous UPLC‐QTOF‐MS‐based metabolomics study also identified hesperetin as a potential marker for GCP (Luo et al. 2019). Hesperetin is known to be generated through the degradation of hesperidin during the aging process (Wang et al. 2016). Studies have shown that hesperidin decreases while hesperetin increases with prolonged aging (Wang et al. 2020, 2018). Therefore, further investigation is needed to evaluate the potential of the hesperidin/hesperetin ratio as a marker of GCP aging duration.

#### 3.4.2 Multivariate Analysis

##### 3.4.2.1 PCA and OPLS‐DA Analysis

**FIGURE 4:**
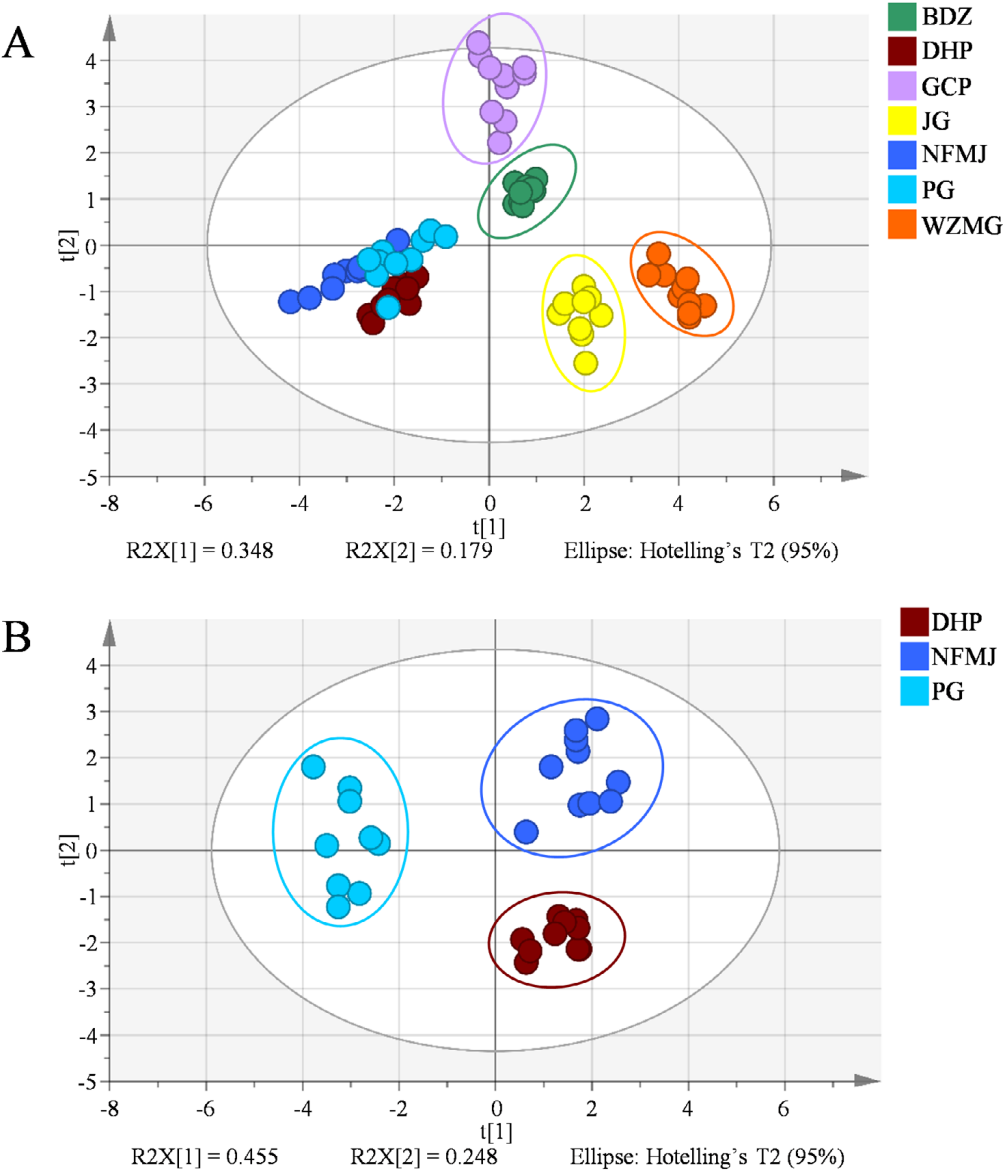
PCA score plots of the seven CP cultivars (A) and a focused comparison among DHP, NFMJ, and PG (B).

**FIGURE 5:**
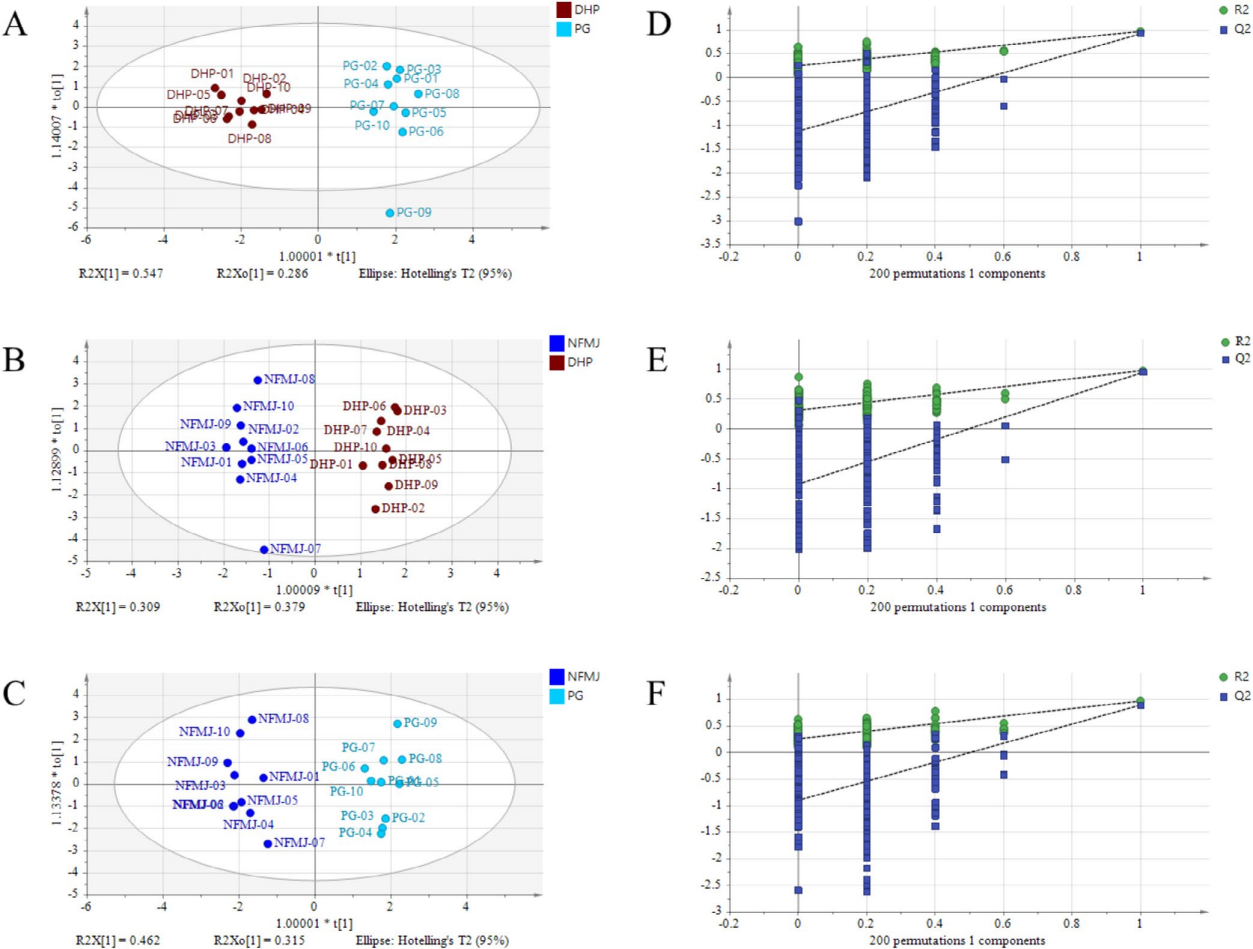
OPLS‐DA score plots comparing DHP, NFMJ, and PG (A–C), and results of the 200‐time permutation tests (D–F).

PCA was performed based on the levels of nine flavonoids to distinguish among the seven CP cultivars. As shown in Figure 4A, the cumulative *R* ^2^ *X* and *Q* ^2^ values were 0.969 and 0.753, respectively, indicating excellent model performance. According to the PCA score plot, BDZ, NFMJ, DHP, JG, GCP, and WZMG were clearly separated without overlap, suggesting distinct flavonoid profiles among these cultivars. Although PG partially overlapped with NFMJ and DHP, its samples clustered relatively tightly. To further resolve the similarities among these three cultivars, a separate PCA was conducted for PG, NFMJ, and DHP, in which each cultivar formed an independent cluster (Figure 4B). This result indicates that notable differences remain among PG, NFMJ, and DHP, despite their relatively similar flavonoid profiles. To further distinguish these three cultivars, three independent OPLS‐DA models were constructed. As shown in Figure 5A–C, the *R* ^2^ *Y* and *Q* ^2^ values of all three models exceeded 0.967 and 0.895, respectively. Furthermore, 200‐time permutation tests demonstrated that none of the models were overfitted, confirming their robustness (Figure 5D–F). These OPLS‐DA results were consistent with the PCA findings and further validated the clear differentiation among PG, NFMJ, and DHP.

The accumulation of flavonoids is influenced by both genetic background and environmental conditions (Su et al. 2023; Wen et al. 2024). In this study, WZMG samples were collected from Zhejiang and Hunan Provinces (China), two geographically close regions with similar environmental conditions. DHP samples were collected from Sichuan Province and Chongqing Municipality (China), which are adjacent and share comparable climates. PG samples were collected from Sichuan and Fujian Provinces (China), which are geographically distant and differ markedly in environmental conditions. The PCA results revealed that WZMG and DHP samples exhibited tighter intra‐group clustering, whereas PG samples were more dispersed. This clustering pattern may reflect environmental variation among the cultivation regions.

##### 3.4.2.2 HCA Analysis

**FIGURE 6:**
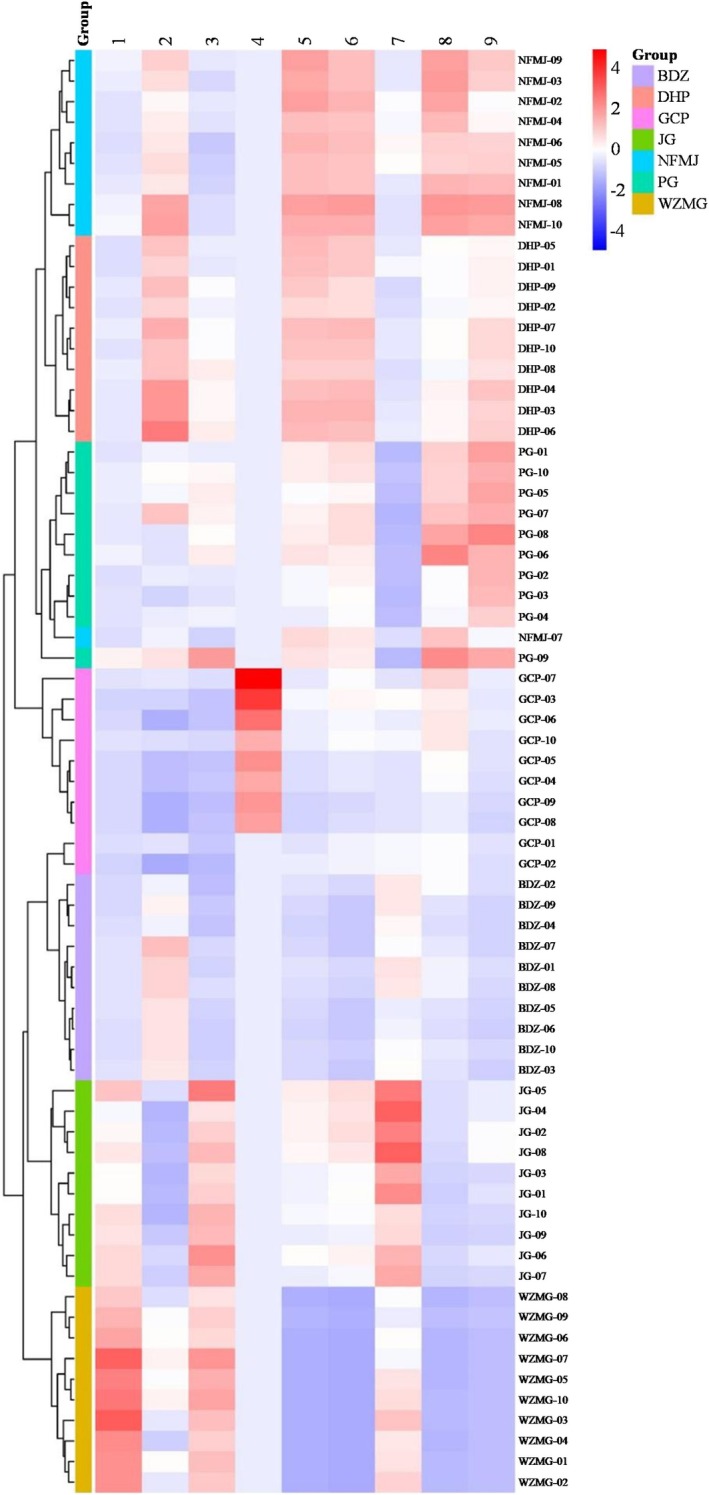
Hierarchical clustering analysis (HCA) heat map and clustering results of the seven CP cultivars based on the contents of the nine target flavonoids. (1) Narirutin, (2) Hesperidin, (3) Didymin, (4) Hesperetin, (5) Sinensetin, (6) Nobiletin, (7) 3′,4′,3,5,6,7,8‐heptamethoxyflavone, (8) Tangeretin, (9) 5‐Demethylnobiletin.

To further validate the discriminative capacity of the nine flavonoids, hierarchical clustering analysis (HCA) was conducted. As shown in Figure 6, samples from the seven CP cultivars formed distinct clusters, consistent with the PCA and OPLS‐DA results. One batch of NFMJ clustered with PG, as also observed in the PCA, highlighting the similarity in flavonoid composition between these two cultivars.

Previous studies quantified five flavonoids using HPLC and combined the results with HCA to distinguish GCP from other CP cultivars (Luo et al. 2018). In the Chinese Pharmacopeia, CP is classified into common CP and GCP. GCP has a characteristic trilobed shape and is traditionally regarded as superior in quality, which contributes to its higher market price. During preliminary field investigations, we found that WZMG was processed into a trilobed shape and fraudulently marketed as GCP. Such artificially processed trilobed samples exhibit similar morphology to real GCP, making it extremely difficult to distinguish them based on their shape and color. In this study, quantitative analysis of nine flavonoids, followed by PCA/OPLS‐DA and HCA, enabled clear differentiation among the seven CP cultivars, including GCP. This method provides a robust tool for cultivar authentication, quality traceability, and market regulation of CP.

Interestingly, among the 10 GCP batches, two (GCP‐01 and GCP‐02) clustered together with BDZ. These two batches had been aged for only 1 and 2 years, respectively, whereas the remaining eight batches had been aged for at least 3 years. This finding suggests that aging significantly alters the chemical profile of GCP and supports the traditional practice of aging GCP for a minimum of 3 years prior to use. Additionally, although GCP and BDZ can be distinguished morphologically before processing, they may become difficult to differentiate once sliced for medicinal use. This further highlights the importance of chemical analysis for accurate identification and quality control.

## 4 Conclusion

In this study, the chemical composition of seven CP cultivars was characterized using LC–MS/MS. Among the 53 identified compounds, nine flavonoids were selected to establish a rapid and accurate UPLC‐based quantitative method. Quantification of the nine flavonoids, combined with PCA, OPLS‐DA, and HCA, enabled clear differentiation among the seven cultivars. Among these cultivars, PG, NFMJ, and DHP exhibited relatively similar chemical profiles. Notably, hesperetin was quantified exclusively in GCP, suggesting its potential as a characteristic marker for identifying this cultivar. Overall, this study provides a valuable reference for quality evaluation, authentication, and further research on CP cultivars.

## Author Contributions

**Danlin Lin:** conceptualization, methodology, data duration, writing – original draft. **Jinju Zhang:** investigation, writing – review and editing. **Mingqi Chen:** investigation, validation. **Chuchu Zhong:** investigation, validation. **Hui Cao:** conceptualization, writing – review and editing. **Zhiguo Ma:** investigation, writing – review and editing. **Menghua Wu:** investigation, project administration, supervision. **Ying Zhang:** conceptualization, resources, funding acquisition, project administration, supervision.

## Funding

This work was supported by the Guangdong Key Lab of Traditional Chinese Medicine Information Technology (2021B1212040007) and the National Famous Traditional Chinese Medicine Expert Inheritance Studio Construction Project (State Administration of Traditional Chinese Medicine [2022] 75).

## Conflicts of Interest

The authors declare no conflicts of interest.

## Supporting information


**Appendix S1:** Representative total ion chromatograms of the seven CP cultivars.

**Appendix S2:** Optimization results for extraction parameters: (A) extraction solvent; (B) extraction method; (C) extraction time; (D) extraction volume.

**Appendix S3:** UV absorption spectra of the nine target compounds in the mixed standard solution and the GCP‐01 sample solution. (A1–A9) mixed standard solution. (B1–B9) the GCP‐01 sample solution.

## Data Availability

The data that support the findings of this study are available from the corresponding authors upon reasonable request.
