# Supplementary figures and images for: Identification of Citri Reticulatae Pericarpium (Chenpi) From Different Cultivars via LC–MS/MS and UPLC Coupled With Multivariate Chemometrics Analysis
Source: Food Sci Nutr. 2026 Mar 6;14(3):e71591. doi: 10.1002/fsn3.71591 (PMC12965897; doi:10.1002/fsn3.71591)

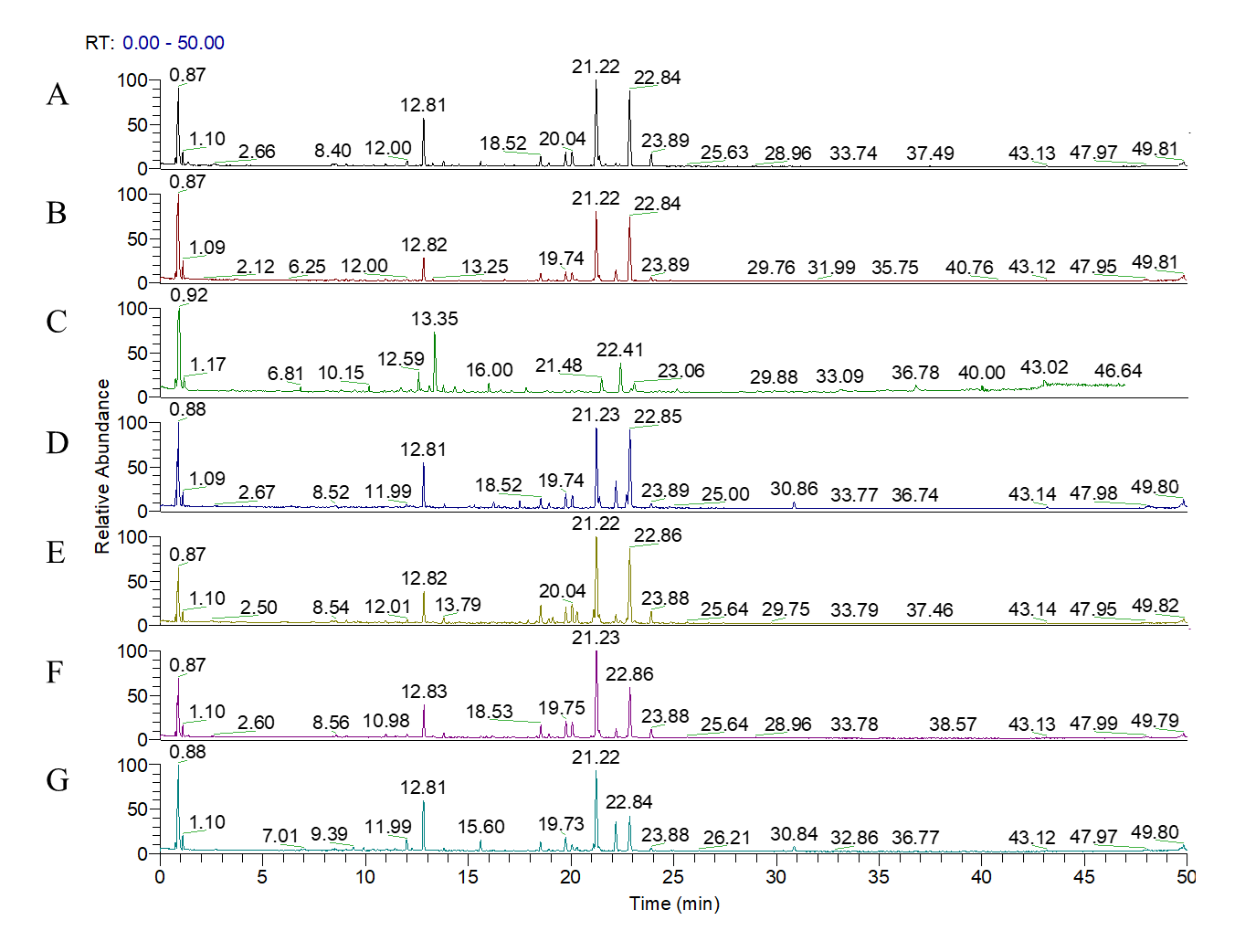

Supplement: Supplementary file 1 — Appendix S1: Representative total ion chromatograms of the seven CP cultivars. [file FSN3-14-e71591-s003.tiff]

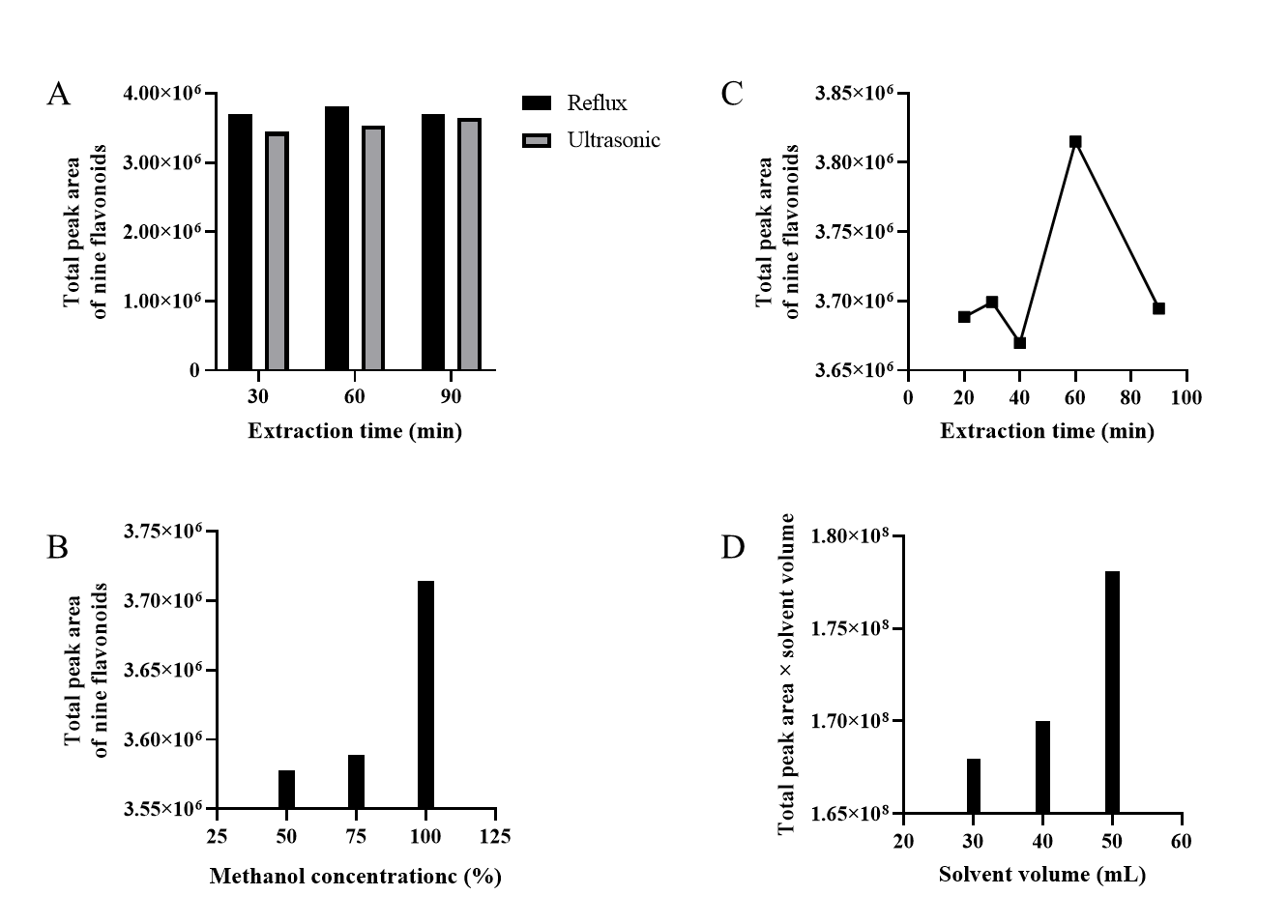

Supplement: Supplementary file 2 — Appendix S2: Optimization results for extraction parameters: (A) extraction solvent; (B) extraction method; (C) extraction time; (D) extraction volume. [file FSN3-14-e71591-s002.tiff]

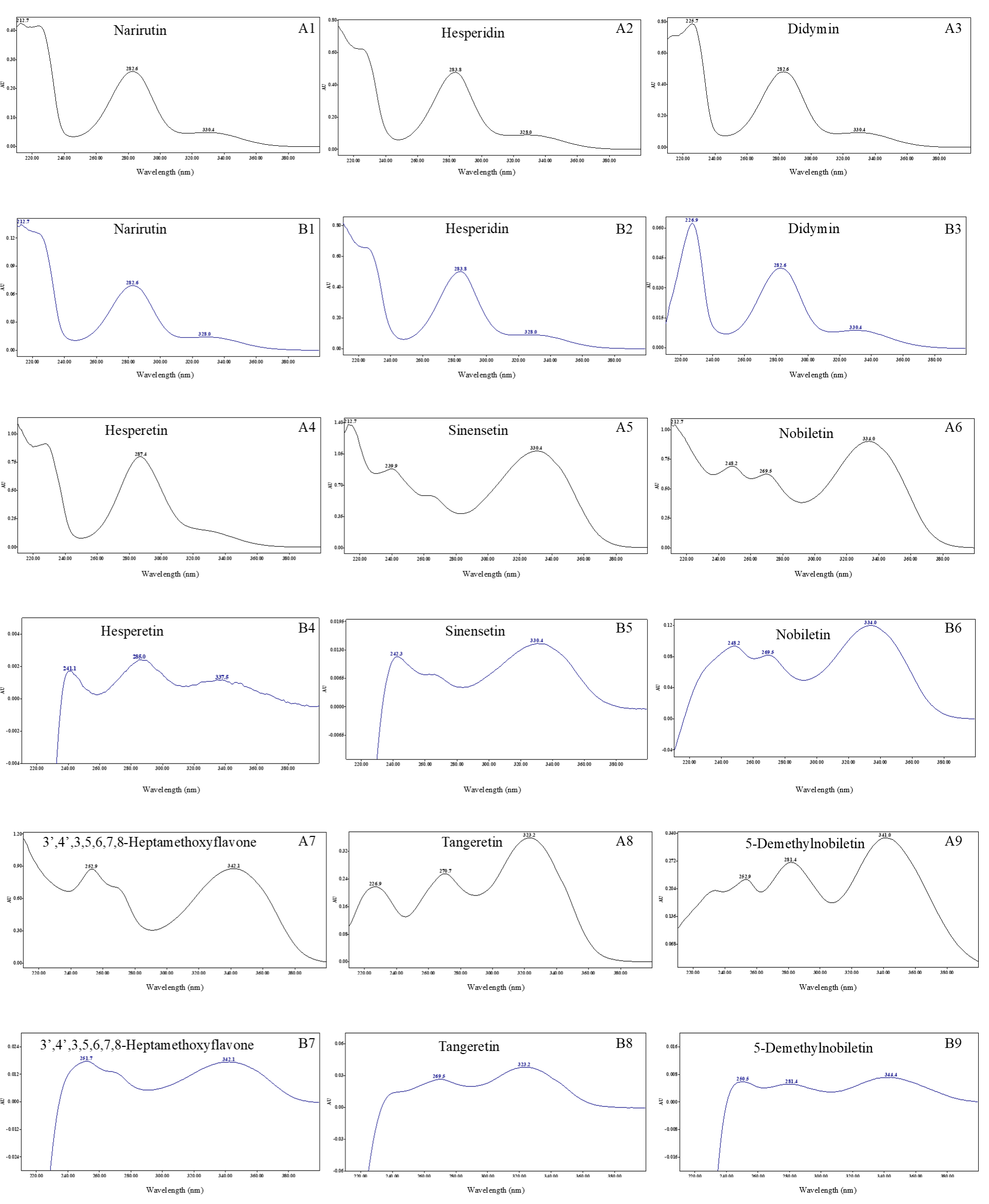

Supplement: Supplementary file 3 — Appendix S3: UV absorption spectra of the nine target compounds in the mixed standard solution and the GCP‐01 sample solution. (A1–A9) mixed standard solution. (B1–B9) the GCP‐01 sample solution. [file FSN3-14-e71591-s001.tiff]
